# Next steps in studying the human microbiome and health in prospective studies, Bethesda, MD, May 16–17, 2017

**DOI:** 10.1186/s40168-018-0596-z

**Published:** 2018-11-26

**Authors:** Rashmi Sinha, Habibul Ahsan, Martin Blaser, J. Gregory Caporaso, Joseph Russell Carmical, Andrew T. Chan, Anthony Fodor, Mitchell H. Gail, Curtis C. Harris, Kathy Helzlsouer, Curtis Huttenhower, Rob Knight, Heidi H. Kong, Gabriel Y. Lai, Diane Leigh Smith Hutchinson, Loic Le Marchand, Hongzhe Li, Michael J. Orlich, Jianxin Shi, Ann Truelove, Mukesh Verma, Emily Vogtmann, Owen White, Walter Willett, Wei Zheng, Somdat Mahabir, Christian Abnet

**Affiliations:** 1 Division of Cancer Epidemiology and Genetics, National Cancer Institute, Bethesda, MD 20892 USA; 2 Comprehensive Cancer Center University of Chicago Medicine and Biological Sciences, Chicago, IL 60615 USA; 3 Departments of Medicine and Microbiology, New York University Langone Medical Center, New York, NY 10016 USA; 4 Pathogen and Microbiome Institute and Department of Biological Sciences, Northern Arizona University, Flagstaff, AZ 86011 USA; 5 Department of Molecular Virology and Microbiology, Baylor College of Medicine, Houston, TX 77030 USA; 6 Clinical and Translational Epidemiology Unit, Massachusetts General Hospital and Harvard Medical School, Boston, MA 02114 USA; 7 Division of Gastroenterology, Massachusetts General Hospital, Boston, MA 02114 USA; 8 Channing Division of Network Medicine, Department of Medicine, Brigham and Women’s Hospital, and Harvard Medical School, Boston, MA 02115 USA; 9 Department of Immunology and Infectious Diseases, Harvard T.H. Chan School of Public Health, Boston, MA 02115 USA; 10 Broad Institute of Massachusetts Institute of Technology and Harvard, Cambridge, MA 02142 USA; 11 Department of Bioinformatics and Genomics, University of North Carolina at Charlotte, Charlotte, NC 28223 USA; 12 Laboratory of Human Carcinogenesis, National Cancer Institute, National Institutes of Health, Bethesda, MD 20892 USA; 13 Division of Cancer Control and Population Sciences, National Cancer Institute, National Cancer Institute, National Institutes of Health, Bethesda, MD 20892 USA; 14 Department of Biostatistics, Harvard T. H. Chan School of Public Health, Boston, MA 02115 USA; 15 Center for Microbiome Innovation, and Departments of Pediatrics and Computer Science and Engineering, University of California San Diego, San Diego, CA 92093 USA; 16 Dermatology Branch, National Cancer Institute, National Cancer Institute, National Institutes of Health, Bethesda, MD 20892 USA; 17 Environmental Epidemiology Branch, National Cancer Institute, Bethesda, MD 20892 USA; 18 Alkek Center for Metagenomics and Microbiome Research, Department of Molecular Virology and Microbiology, Baylor College of Medicine, Houston, TX 77030 USA; 19 Cancer Epidemiology Program, University of Hawaii Cancer Center, Honolulu, HI 96813 USA; 20 Department of Biostatistics, Epidemiology and Informatics, University of Pennsylvania Perelman School of Medicine, Philadelphia, PA 19104 USA; 21 School of Public Health and Department of Preventive Medicine, School of Medicine, Loma Linda University, Loma Linda, CA 92350 USA; 22 Westat, Rockville, MD 20850 USA; 23 Institute for Genome Sciences, University of Maryland School of Medicine, Baltimore, MD 21201 USA; 24 Departments of Epidemiology and Nutrition, Harvard T.H. Chan School of Public Health, Boston, MA 02115 USA; 25 Division of Epidemiology, Vanderbilt University Medical Center, Nashville, TN 37232 USA

**Keywords:** Microbiome, Prospective, Cohort, Biospecimen, Cancer, Epidemiology

## Abstract

The National Cancer Institute (NCI) sponsored a 2-day workshop, “Next Steps in Studying the Human Microbiome and Health in Prospective Studies,” in Bethesda, Maryland, May 16–17, 2017. The workshop brought together researchers in the field to discuss the challenges of conducting microbiome studies, including study design, collection and processing of samples, bioinformatics and statistical methods, publishing results, and ensuring reproducibility of published results. The presenters emphasized the great potential of microbiome research in understanding the etiology of cancer. This report summarizes the workshop and presents practical suggestions for conducting microbiome studies, from workshop presenters, moderators, and participants.

## Introduction

Interest in the role of the microbiome in health has been increasing, as evidenced by the May 2016 announcement of the National Microbiome Initiative (NMI) (https://obamawhitehouse.archives.gov/blog/2016/05/13/announcing-national-microbiome-initiative), to advance the use of microbiome science in health care, food production, and environmental restoration. The National Institutes of Health (NIH) invested $20 million in microbiome research as part of the NMI in fiscal years 2016 and 2017. NIH is focusing on multi-ecosystem comparison studies and the design of new tools to explore and understand microbiomes.

To date, few prospective epidemiological studies investigating the role of the microbiome in human health have been published. Epidemiological studies from which we can draw inferences will require accurate and reproducible assays, knowledge of potential factors affecting the microbiome, an understanding of its metabolic functions, and more. We will need to replicate findings across multiple populations and, ideally, pool data from many different study designs. Variation may occur at each step in the research pipeline: sample collection, storage, DNA extraction, polymerase chain reaction (PCR) amplifications, DNA sequencing, bioinformatics, and statistical analyses.

## Meeting goals and objectives

To prepare researchers to conduct epidemiologic studies, the NCI’s Metabolic Epidemiology Branch (MEB) in the Division of Cancer Epidemiology and Genetics (DCEG) and the Epidemiology and Genomics Research Program (EGRP) in the Division of Cancer Control and Population Sciences (DCCPS) held a two-day workshop, “Next Steps in Studying the Human Microbiome and Health in Prospective Studies,” in Bethesda, MD, from May 16–17, 2017. More than 200 participants from academia, government agencies, and industry attended the workshop.

The workshop began with an overview of the field, including the fundamental questions facing researchers. It then focused on five key areas:

- Optimizing sample collection for multi-omics analyses;
- Minimizing variation in sample and data processing;
- Optimizing statistical methods for analyzing microbiome data;
- Designing epidemiologic studies for microbiome research; and
- Defining reporting and data sharing standards.

Additionally, speakers presented information about the NCI extramural microbiome epidemiology grants portfolio, and ongoing NCI intramural microbiome studies. Here, we present a synopsis of the discussions and highlight important questions and suggestions for future work.

## Overview: fundamental questions

Drs. Stephen Chanock (Director, DCEG, NCI) and Kathy Helzlsouer (Associate Director, EGRP, DCCPS, NCI) welcomed the attendees and tasked them to fulfill the goals of the meeting, which were to present current findings, discuss issues related to rigor and reproducibility, and provide suggestions for carrying out microbiome studies in prospective cohorts. Next, four presenters spoke about the current state, future potential, and fundamental questions of microbiome research.

In his talk that set the stage for the conference, Dr. Rob Knight said that billions of dollars go to human genome sequencing, but humans are more microbe than human. Microbial cells outnumber human cells slightly and microbial genes outnumber human genes dramatically. The Human Microbiome Project (https://hmpdacc.org/) collected ~ 4.5 trillion bases of DNA (1500 times the human genome). Importantly, the genes in our microbiome are not fixed at birth, are controlled by lifestyle choices, and encode many of our unique metabolic functions.

We can now create a reference map of the microbiome during processes ranging from normal infant development to the events that occur following a fecal microbiota transplant using principal coordinates analysis, a method to explore and visualize similarities or dissimilarities of data based on evolutionary distances between microbes in pairs of samples. A major challenge is understanding which of the many diseases, including various forms of cancer and cancer risk factors (e.g., obesity and inflammatory bowel disease), are detectable as different locations on this map. Data collected by more than 10,000 citizen scientists in the American Gut Project [1] may help fill in this map, but studies of well-defined and carefully phenotyped cohorts, who are monitored over time, will provide even greater value.

Dr. Curtis Harris described his ongoing studies focused on the microbiome of lung cancer. His analysis demonstrated that the cancerous lung is characterized by microbial dysbiosis and harbors a distinct group of bacteria inside the tumor cells depending upon the lung cancer type. The types of bacteria inside the tumor cells vary depending on the lung cancer type. These distinct bacterial genera were most abundant in squamous cell carcinomas with TP53 mutations. He also discussed the need for bacterial isolates to identify specific strains that may differ in their biological and pathological activity.

Dr. Helzlsouer noted that elucidating the complex association between microbiomes and cancer risks will require a rigorous and reproducible stepwise approach that progresses from small-scale methodologic studies to large-scale population-based research [2]. It will be critical to study the metabolic products of the microbiome that are absorbed at the tissue level and that circulate systemically.

Reproducibility of methods within and between laboratories is critical. Studies have shown that there is marked variation between laboratories in results for diversity. Laboratories also use different methods for sample collection and processing [3–5]. In addition to laboratory sources of variation, many factors may affect reproducibility of results. Mouse studies have demonstrated immense variability in residential microbes due to factors such as diet, housing, and stress [6]. Understanding the many factors that influence the microbiome is necessary to guide research decisions, such as the type(s) of specimens collected, as well as frequency of the collections and accompanying metadata.

Dr. Helzlsouer noted microbiome research is an exciting and interesting area of study, but methodological challenges suggest an adage written by Richard Harris: [7] “…to speed the development of medicine, biomedical science should actually slow down. This means taking of fewer projects and doing them more carefully.”

Dr. Martin Blaser highlighted five key points:

1. *Microbiota participate in oncogenesis.* It has become clear over the past 25 years that persistent *Helicobacter pylori* (*H. pylori*) colonization is associated with an increased risk of adenocarcinoma affecting the gastric corpus and antrum. Now attention is focused on the roles of microbiota in colon cancer, as well as estrogen-driven cancers of the breast, ovary, and endometrium.
2. *Risk may begin early in life.* Consistent with the model of Hepatitis B infection and liver cancer, there is evidence that the interaction early in life between host and microbes affects the risk of gastric cancer. This is remarkable since these cancers typically present in the seventh and eighth decades of life.
3. *Microbiota are changing.* Important information in recent years indicates that our microbiota is becoming less diverse and some taxa are becoming extinct in some people.
4. *There may be multiple potential mechanisms.* Mechanisms implicated in microbial oncogenesis include persistent inflammation and genotoxicity. Furthermore, given the role of the gut microbiota in modulating serum estrogen levels, an enzyme that affects the conjugation of estrogens may be pro-oncogenic. Parallel mechanisms may also be relevant to androgen-driven neoplasia.
5. *Harnessing knowledge of the microbiota may lead to new preventive strategies, diagnostics, and, potentially, new treatments.* Specific interactions, such as checkpoint inhibitors, between microbiota and the immune system are potential targets for treatments. But identifying particular taxa at the species and strain level, as well as their metabolic pathways and metabolites, presents another frontier for developing diagnostic, preventive, and therapeutic approaches.

## Optimizing sample collection methodology and quality control (QC) standards

Dr. Rashmi Sinha presented data on fecal collection methods that could be implemented under typical epidemiologic cohort field conditions. She investigated six methods on fecal samples gathered in four studies from 132 individuals [3, 5, 8, 9]. The methods were no additive, RNA*later*, 70% and 95% ethanol, card-based preservation (fecal occult blood test [FOBT] 16S rRNA gene or Flinders Technology Associates [FTA]) cards), and fecal immunochemical testing (FIT) tubes.

Reassuringly, the major source of variation of fecal microbial profiles using 16S rRNA gene sequencing was between individual persons, followed by between sampling methods and lengths of time at ambient temperature.

All six methods delivered excellent reproducibility. Except for no additive and 70% ethanol, they all delivered good or excellent stability over 4 or 7 days at ambient temperature. Compared to the “gold standard” (rapidly frozen, no additive samples), the most accurate results were found with 70% and 95% ethanol, FOBT/FTA, and RNA*later*. However, these collection methods differed in the relative abundances of various bacteria. This finding has significant implications for future epidemiologic studies. For adequate power to detect disease associations with specific microbial taxa, microbiome data will have to be pooled across multiple studies. If individual studies collect fecal samples using different methods, conducting pooled analyses or meta-analyses may not be possible. It is important for different prospective studies to coordinate and collect fecal samples using at least one common method in addition to the method of their choice.

Dr. Sinha also presented shotgun sequencing and metabolomics data. For shotgun sequencing, FOBT, FIT, and RNA*later* provided robust results. For fecal metabolomics, 95% ethanol or FOBT demonstrated stability. Thus, future epidemiologic studies should collect feces using 95% ethanol or FOBT if interested in studying fecal metabolomics [4].

Dr. Emily Vogtmann provided insights into temporal variability and the impact of different collection methods on oral microbiota. On oral samples collected over 10 months from 40 individuals, she calculated intraclass correlations (ICCs) for specific microbial diversity metrics. In general, ICCs were relatively high, particularly for alpha diversity metrics Chao1 and observed species, but ICCs decreased for relative abundances at the phylum level. Researchers could use these estimates to determine sample size requirements. Collecting multiple samples over time would decrease required sample sizes, particularly for metrics with lower ICCs.

In two studies, Dr. Vogtmann found that Scope mouthwash did a better job preserving the rank order of participants for the relative abundance of the top phyla and for alpha and beta diversity estimates, compared to using saliva collected in the OMNIgene ORAL kit. However, the Scope mouthwash samples had some distinct microbial characteristics compared to the OMNIgene ORAL samples. Like the findings from fecal samples, future studies should compare oral microbial metrics within one sample collection type.

Dr. Joseph Russell Carmical discussed different types of reference materials (RM). RM is any stable, abundant, and well-characterized specimen used to assess the quantitative and/or qualitative validity of a measurement process. Metagenomic analyses commonly use whole cell RMs. They fall into three categories: environmental, pure microbial isolates, and in vitro models of microbial ecosystems. Each has its pros and cons.

Environmental samples must closely resemble the complexity of the microbial community being evaluated, but complexity is difficult to characterize. Furthermore, changes in the microbial makeup over time result in variability in the RM, which is not suitable for longitudinal studies. Pure microbial strains and mock communities are characterized more easily and are abundant, but they lack the complexity of the environmental samples. In vitro models are cultivated in a controlled environment (bioreactors); thus, they approach the complexity of an environmental sample but make characterization easier. However, one cannot truly recapitulate the complexity of an environmental sample. Also, batches created in bioreactors may vary from each other. The three types of RMs can be used in combination, if appropriate for the experimental design.

In break-out sessions, participants also discussed what sampling approaches to use. Several fecal sample collection approaches (FOBT, RNA*later*, 95% ethanol) provide high-quality DNA for 16S rRNA gene and metagenomics, but some sample collection approaches are not as useful for fecal metabolomics or metatranscriptomics.

It would be ideal to collect samples using more than one preservation method if financially feasible. Implementing a dual collection approach (using at least one common collection method) even on a subset of participants would allow for comparisons across cohorts. But the scientific questions need to guide the decisions about what approaches to use. More information on the effects of long-term storage is needed. Also, a standard protocol for adding samples with synthetic DNA should be considered to allow for monitoring loss during sample processing.

## Variation due to extraction, amplification, sequencing, and bioinformatics

Dr. Curtis Huttenhower presented on the Microbiome Quality Control (MBQC) project (http://www.mbqc.org/) [10, 11], a collaborative effort of the NCI and many individual labs to identify and quantify sources of biological and technical variation in human microbiome research. An initial baseline study, the MBQC-base, recently characterized the effects of three typical steps in 16S rRNA gene surveys of human stool samples: nucleotide extraction, sequencing, and data analysis. The MBQC-base, modeled on previous efforts such as the Microarray Quality Control (MAQC) [12] and Sequencing Quality Control (SEQC) [13] projects, replicated, blinded, and distributed a small set of fecal biospecimens to 15 data generation laboratories. The labs put their resulting amplicon sequencing data at MBQC Data Analysis and Coordination Center (DACC) (http://ihmpdcc.org/MBQC/), and nine bioinformatics groups re-blinded and analyzed the data. The study assessed the sources and extent of measurement accuracy and variability of more than 16,500 profiles—approximately three times the amount of amplicon data from the Human Microbiome Project.

The team analyzed several aspects of microbiome data generation and analysis protocols. Fortunately, for microbiome population studies, differences between individuals and biospecimens types were typically the largest. However, DNA extraction and sample handling environment contributed substantially to the variability. Other protocol variables and computing differences had smaller effects. Using controls—artificial communities as positive controls and extraction reagents as negative controls—helped identify differences in environment-specific contamination, nucleotide extraction, and bioinformatic classification. A more systematically designed MBQC-II is currently being planned, but will require financial support. The new study would test multiple experimental designs and inform researchers carrying out comparable microbiome studies across laboratories and cohorts.

In her talk, Dr. Diane Smith Hutchinson noted that researchers are increasingly including metagenomic characterization of the microbiome in ever larger studies of how environmental and genetic factors interact to influence disease susceptibility.

The analytic outputs of metagenomic shotgun sequencing include high-resolution descriptions of bacteria, archaea, and sometimes DNA viruses, as well as gene content information (e.g., metabolic potential). Due to the nature of the data generated, the resulting primary outputs are represented by sparse and zero-inflated compositional tables. This is a problem when assessing the statistical relationship between microbiome metrics and clinical covariates. Furthermore, longitudinal sampling adds another layer of complexity for statistical modeling. Researchers have tried to overcome these challenges, by evaluating the data in a cross-sectional manner, reducing dimensionality by clustering samples into groups, employing linear (or nonlinear) mixed-effects models, and performing conditional logistic regressions with summary metrics created from microbiome data.

Dr. J. Gregory Caporaso presented the Quantitative Insights Into Microbial Ecology 2 (QIIME 2) microbiome bioinformatics platform (https://qiime2.org) [14], a complete re-write of the widely used QIIME 1 software. He focused on one novel feature of QIIME 2: automated, decentralized provenance tracking of all the bioinformatic steps of an analysis, including methods applied and parameters used. It also tracks information on the software environment where an analysis was run. QIIME 2 is a major advance toward improved reproducibility of bioinformatics analysis.

Dr. Caporaso described other advances in QIIME 2 as well, including improved sequence quality control, a focus on analysis of sequence variants rather than OTU clusters for improved taxonomic resolution, and new machine learning and alignment-based approaches for taxonomic assignment of sequences. QIIME 2 also provides interfaces for different types of users, including a prototype graphical user interface for end users without advanced computer skills, a command line interface for power users, an application programmer interface (API) for data scientists and programmers, and a web interface for viewing QIIME 2 results on systems that do not have QIIME 2 installed. Finally, QIIME 2 is based on a plugin architecture that allows third-party developers to easily make their bioinformatics software available to users through QIIME 2. A community of software developers around the world are writing QIIME.

In the break-out sessions, participants discussed the importance of laboratories using automation when processing samples, to avoid variation due to handling. They stressed the importance of including both negative and positive controls at all stages of sample processing. Participants considered knowledge sharing to be important to develop standardized protocols, especially when methods did not work. Data analysis methods need to be transparent and reproducible; using a bioinformatics platform such as QIIME 2 that provides automated data provenance tracking helps. Furthermore, a data repository that supports long-term archiving of primary microbiome data (i.e., sequence data) as well as sample and study metadata, and allows users to easily deposit and retrieve these data in common formats is currently an unmet need in the field.

## Statistical considerations in the design, analysis, and interpretation of microbiome studies

Dr. Anthony Fodor reviewed the relationship between biological variability, technical artifacts, and reproducibility in microbiome studies. He reminded the group that in any biological experiment, reproducibility is possible when there is a consistent signal that is larger than the sum of the measurement error and biological variability. As a result of statistical methods work, we are beginning to understand how each component in processing of the microbial community impacts technical reproducibility. While different sequencing runs on the same platform tend to introduce only small amounts of technical variability, techniques used for extraction of microbial DNA have a profound impact on the composition of the microbial community [15]. PCR steps can introduce substantial bias; interactions between different taxa in PCR reactions [16] can be difficult to predict ahead of time.

Dr. Fodor noted that standardizing techniques can reduce the impact of technical artifacts, but reproducibility also requires an effect size that is larger than biological stochasticity. A strong association between a disease and the microbiome would be easier to reproduce than a weak association. Conditions such as colorectal cancer and liver cirrhosis clearly have stronger associations with microbial community composition than obesity [17], and this presumably explains why there is a more robust reproducible signal across studies for colorectal cancer [18, 19] than for obesity [17, 20, 21]. We have increasing evidence for a common signal of dysbiosis across multiple diseases, but it is unclear how much of this signal is due to shared pharmacology [22, 23] rather than the underlying disease state.

While 16S rRNA gene sequencing provides information about microbial composition, shotgun metagenomic sequencing provides information about microbial genes and pathways, explained Dr. Hongzhe Li. Knowing the taxonomic composition and the gene composition can be important to understanding human diseases. Based on the uneven coverage of the sequencing reads from the origin and terminus of bacterial replication, shotgun sequencing data also provide important insights into the microbial replication rates and growth dynamics [24]. All these microbiome features have been shown to be associated with various human diseases or treatment outcomes.

Features of the microbiome can be treated as outcome, exposure, or covariate. When treated as the outcome, the statistical issues focus on testing the effect of the exposure on the overall microbial composition or on each of the taxa. When treated as a covariate in clinical outcomes, the microbiome can serve as a moderator of the treatment effect in a regression analysis framework. Since the data obtained from 16S rRNA gene or shotgun metagenomic sequencing can only provide relative abundance information, the microbial abundance data are compositional with a unit sum. When the microbial relative abundances are used as covariates in regression analysis, the compositional nature of the data has to be accounted for in order to achieve the subcompositional coherence [25, 26] and to reduce false positive results.

The microbiome may serve as an important mediator of treatment effects. With appropriately designed studies, such as randomized experiments or longitudinal studies, researchers can apply methods from the causal mediation analysis literature to quantify the mediating effect of the microbiome. Such mediation analyses link treatment to outcomes and identify the subcomposition of the microbial community that serves as an important mediator. However, existing mediation analysis methods need to be extended to account for the high dimensional and compositional nature of the microbiome data and to adjust for confounding factors [27]. It is important to perform rigorous sensitivity analyses for unmeasured confounding.

Dr. Jianxin Shi discussed using data from prospective studies for estimating the overall contribution of the human microbiome on the risk of developing a complex disease. Although data from large-scale prospective studies are not yet available, Dr. Shi cited BMI and American Gut Project data as an example [28]. Based on a linear mixed model and the 4001 OTUs (average relative abundance > 0.5%), the relative abundance data explained 27.0% (standard error (S.E.) 2.1%) of BMI variance and presence/absence data explained 34.2% (S.E. 2.7%).

Dr. Shi also discussed the potential confounding effects of population stratification when testing for an overall association using beta-diversity analysis in large-scale studies. In genome-wide association studies, population stratification is typically controlled by principal component analysis based on “beta-diversity matrix” using genome-wide SNP data. Similarly, beta-diversity tests of the microbiome may capture population substructure. When such population structure is associated with the disease risk, testing for the overall association using the beta-diversity test will cause spurious associations with disease. He suggested that such confounding may be investigated after obtaining host genetic data in large-scale studies.

In the breakout session, participants suggested that statisticians and computational biologists should be consulted at all stages of study planning as their input is valuable for determining sample sizes and conducting statistical analyses. Studies must be designed appropriately to ensure that the results will allow for substantive interpretation and conclusions, especially since the results will need to be adjusted for multiple comparisons thereby lowering the minimum *p* value required for statistically significant findings.

## Epidemiologic study design

Owing to a lack of studies with fecal samples collected in prospective cohort studies, most microbiome studies to date have used a cross-sectional case-control design. Although these studies have provided insight into the differences between the microbiome of those with and without cancer, they are unable to evaluate how the microbiome may be related to the causes of cancer. To address this problem, we need to expand our existing cohorts and set up new ones.

Dr. Wei Zheng described Vanderbilt University Medical Center’s (VUMC’s) three large prospective cohort studies: the Shanghai Women’s Health Study (SWHS), the Shanghai Men’s Health Study (SMHS), and the Southern Community Cohort Study (SCCS). The studies are following about 222,600 participants for cancer incidence and cause-specific mortality. Using oral samples (saliva or mouth-rinse) collected from these participants, multiple studies have evaluated the association of the oral microbiome with the risk of cancers of the colon/rectum, upper aero-digestive tract, lung, pancreas, and stomach, as well as type 2 diabetes and obesity. VUMC investigators have also collected stool samples from approximately 16,000 healthy participants since 2014 to study the association of chronic disease and gut microbiome.

Based on a report by Dr. Loic Le Marchand, preliminary data from 2771 participants of the Multiethnic Cohort (MEC) study showed that race/ethnicity (self-reported or based on genetic ancestry) was associated with small but significant differences in gut microbial community. MEC includes 215,000 well-characterized, older African Americans, Latinos, Japanese Americans, Native Hawaiians, and European Americans in Hawaii and California. A pattern of less alpha-diversity (Shannon index) was observed for women and Japanese Americans. Dr. Le Marchand also reviewed data on the optimization of a home stool collection protocol with immediate sample preservation in RNA*later* and on the stability of the samples during over-night shipping and in long-term storage at − 80 °C.

Dr. Michael Orlich reported that one potential source of fecal and oral samples may be the 70,000 living cohort members of the Adventist Health Study-2 (AHS-2). Response rates from a pilot collection suggest that researchers may be able to collect 32,000 samples. AHS-2 has a cohort of approximately 96,000 Seventh-day Adventist adults living in the USA and Canada. One-fourth of the cohort is African American. Notably, a high proportion of AHS-2 participants are vegetarians: 8% are vegan, 29% are lacto-ovo-vegetarian, 10% are pesco-vegetarian, and 5% are semi-vegetarian (very low quantity of meat and fish consumption).

Dr. Habibul Ahsan reported that a preliminary study of the HEALS cohort (http://www.urb-bd.org/ResearchProjects/ResearchProjects) in Bangladesh showed that the use of antibiotics influenced mortality, suggesting that the microbiome plays a significant role in determining the health outcomes in this low-resource community. Research has also shown that the relative abundance of different taxa of the gut and oral microbiome varied between the Bangladesh and the US population. Dr. Ahsan outlined his plans for assessing gut and oral microbiome in the Chicago COMPASS cohort (http://compass.uchicago.edu/) focusing on urban health disparity.

Dr. Andrew Chan reported data on men in the Health Professionals Follow-up Study (HPFS) showing that sequencing of mailed self-collected stool specimens using a fixative comprised of either 95% ethanol or RNA*later* provided comparable metagenomic and metatranscriptomic data as stool that was collected and immediately frozen in − 80 °C [29]. A lifestyle and dietary validation study nested within the HPFS found a high within-person stability of the metagenome compared to the stability of the metatranscriptome over 24 to 72 h and over 6 months. In 2018, Dr. Chan and colleagues launched an effort to collect stool samples (using 95% ethanol and Omni-Gut, a commercial fixative developed by DNAGenotek) from 25,000–35,000 participants of the MICRObiome Among Nurses Study (MICRO-N), part of the Nurses’ Health Study II.

The different presentations demonstrated the ongoing work to collect samples from prospective cohorts. It will be important for the different researchers to coordinate and use one common collection method so that data can be pooled or meta-analyzed in the future.

## Reporting and data sharing guidelines

Dr. Owen White noted that sharing analytical methods is now common in the genomics field for several reasons. It promotes collaboration, which is particularly important to the career development of young investigators. Also, publicly funded research should rightfully be available to everyone. *Microbiome’s* policy is that authors should make available the software and scripts they used to generate their data, so others can replicate all published bioinformatics and statistical analyses. The NIH Genomic Data Sharing Policy (https://osp.od.nih.gov/scientific-sharing/genomic-data-sharing-faqs/?pdf=10976) requires that all new, NIH-funded genomic data projects, which include microbiome studies, use transparent data sharing methods to ensure widespread access to completed study data.

Dr. Christian Abnet reminded workshop participants that the publication of microbiome exposure studies embedded in epidemiologic studies are in their infancy, and many are completed by research groups with little prior work in human populations. Transparent data reporting improves peer-review, editorial decision-making, and replication of study findings [30]. With novel bioinformatic and statistical methods being used on existing data, having data be freely available may also lead to novel discoveries [31]. Furthermore, drawing strong inferences from observational studies requires multiple independent examinations of the same hypothesis with subsequent meta- or pooled analyses. Pooling multiple observational studies will inform those results that may be most amenable to translational or interventional studies.

Extensive experience from other fields, including clinical trials and observational epidemiology with Consort and STROBE guidelines (https://www.strobe-statement.org/index.php?id=strobe-home), respectively, have led to many journals adopting standard reporting guidelines. EQUATOR (Enhancing the QUAlity and Transparency Of health Research) Network (http://www.equator-network.org/reporting-guidelines/) has a wide selection of reporting guidelines.

To encourage researchers to document and share their analytic choices, journal editors should require them to use and share electronic notebooks or other methods for documenting the exact bioinformatic and statistical methods. Such functionality can be embedded in updated versions of popular microbiome processing pipelines. Researchers, funders, and publishers should jointly tackle the challenges of data sharing. Because the microbiome is so sensitive to lifestyle and environment, careful assessment of potential confounding factors will be a crucial component of future studies.

## NCI extramural/intramural grants, resources, and collaborative opportunities

During this session, NCI representatives described NCI resources available for conducting microbiome studies and opportunities for collaborating with NCI investigators.

Dr. Gabriel Lai explained that grants are primarily assigned to one of four extramural divisions: Division of Cancer Biology, DCCPS, Division of Cancer Prevention, and Division of Cancer Treatment and Diagnosis. For fiscal year 2014 to the present, DCCPS held fewer than 25% of NCI grants with a microbiome component but provided nearly 40% of NCI funds.

DCCPS supported 18 microbiome-related grants since 2014. These grants consisted of R01s, R03s and exploratory R21s (research grants), larger P01s (program project grants), and U01 s/UM1s (cooperative agreements). Most of the grants evaluated the microbiome of the gut or oral cavity, or multiple sites for purposes of comparison. Many grants are part of existing studies, often large prospective cohorts, indicating NCI’s recognition of the importance of leveraging existing studies’ infrastructure. Separately, a few grants have been supported via the Funding Opportunity Announcement “Core Infrastructure and Methodological Research for Cancer Epidemiology Cohorts” which requires the collection of microbiome samples as an element of the grant.

Dr. Abnet began with an overview of NIH’s Intramural Research Program (IRP). It aims to complete long-term, high-impact science, including a variety of microbiome studies. Microbiome research at DCEG currently focuses on standardization of methods, microbial communities in cancer etiology, microbial communities across the carcinogenic process, microbial communities and cancer-associated exposures, and developing cohorts that collect optimal biosamples for microbiome hypotheses. DCEG seeks to conduct prospective studies on the microbiome and disease risk while also building quality control samples, methods, and tools for intramural and extramural research.

## Next steps

While emphasizing that we have made substantial progress, especially with sample collection methodology, Dr. Sinha focused on what we can do now to advance microbiome research. The table below (updated since her presentation) describes current collection methods for fecal samples:

**ᅟ:** ᅟ

Collection method	Analytical method			
	16S rRNA gene	Shotgun sequencing	Transcriptomics	Metabolomics
No additive	Poor	Not tested	Not tested	Not tested
70% ethanol	Poor	Not tested	Not tested	Not tested
95% ethanol	Good	Fair/good	Being analyzed	Good
FOBT	Good	Good	Being analyzed	Fair/good
FIT	Good	Good	Being analyzed	Poor
RNA*later*	Good	Good	Good	Failed GC/MS

For adequate power, researchers will likely need to analyze data from multiple studies jointly. However, conducting pooled analyses or meta-analyses may not be possible if individual epidemiologic studies use different collection methods. It is, therefore, important for prospective studies to coordinate and collect fecal samples using at least one common method in addition to the method of their choice. Dr. Sinha urged participants to invest in collecting fecal and oral samples in cohorts now since it will take at least 5 to 10 years to observe health outcomes, and standardize the information participants provide at the time the samples are collected.

DCEG is developing QC standards for use across different cohorts and studies. NCI will supply aliquots of these standards to each cohort study so the standards can be analyzed with actual samples. No less important, Dr. Sinha reminded the audience about the need to retain negative controls or blanks when collecting fecal and oral samples. Including blanks in the sample set at the different stages of the pipeline is also critical.

Dr. Heidi Kong discussed the significant bias that can be introduced during extraction, the importance of identifying potential sources of contamination, and the importance of sufficiently validating methods of extraction [32, 33]. Some of the challenges of PCR amplification are primer selection that is based on microbes of interest (e.g., specific hypervariable regions of the 16S rRNA gene for bacteria; other regions for targeting fungi) and PCR conditions that can be a source of variability. Outlining best practices for PCR conditions and including mock communities and negative controls would improve consistency of PCR amplification across the field.

Decisions concerning common sequencing issues (amplicon versus shotgun metagenomic sequencing, platforms, sequencing depth, and reproducibility) depend on specific research questions, budget, and sequencing platforms. Important considerations for bioinformatics include validating findings by comparing different pipelines and databases, documenting analytical pipelines and parameters, and expanding existing databases with curated strains.

Dr. Walter Willett reviewed the design of studies of microbiome and cancer. In studies of cancer etiology, both case-control and prospective studies have been informative, but their validity depends strongly on the exposure. Case-control studies have been useful when the contrasts in exposure and relative risks are high, such as in comparing smokers with never smokers, heavy alcohol consumption with abstainers, or those with positive serology for HPV infection to seronegative individuals. However, for many other relationships, the results of case-control studies can be misleading due to combinations of selection bias, reporting bias, and reverse causation. These sources of bias will be particularly problematic when the range of exposures and relative risks are more modest, which is expected for common exposures and behaviors related to diet, physical activity, reproductive practices, and sun exposure. Selection bias has become a major problem in population-based studies in the USA because participation rates for controls are now often less than 60%. Because those who participate are likely to be more health conscious and because participation rates of cases remain relatively high, this may seriously distort associations [34]. Recall bias is also a risk and can readily occur when people have been affected by a serious illness. Reverse causation, an effect of disease on exposure, can also be present in case-control studies of cancer; the disease or its treatment can readily cause weight loss and changes in diet, physical activity, sun exposure, and bowel habits. Reverse causation is especially problematic when using biomarkers of exposures because they will be affected by behavioral changes, the disease process, or treatments.

In case-control studies of the fecal microbiome and cancer, dietary factors can modify the microbiome, selection and recall bias will be major concerns, and the presence of disease could change the microbiome directly or indirectly. Because of the likelihood of these biases, or even just their potential, traditional case-control studies of the microbiome and cancer will at best be suspect and at worst seriously misleading. To collect reliable evidence on the microbiome and cancer, we need to establish biorepositories for human samples of feces (collected in a similar manner) and other collections to serve as a basis for nested case-control studies over the next several decades. Animal and human studies, using short interventions and cross-sectional designs, have documented important effects of diet on the fecal microbiome [35, 36]. Thus, studies of the microbiome and cancer need to assess participants’ diets through repeated food frequency or recalls. The method and timing of dietary assessments in relation to sample collections need to be considered. It would be helpful to collect other data during biospecimens collection as well, such as medication, fecal consistency, or probiotic use.

## Conclusions

The human microbiome plays key roles in human health. Workshop participants enthusiastically concluded that microbiome research needs to continue, advance, and expand. The field has advanced in optimizing biological sample collection, processing, and storage; however, additional methodologic work is required. Some cohorts have already collected samples for microbiome studies. But, ideally, cohort studies will use one common collecting method, as well as a method of their choice to help ensure future pooled analyses. Furthermore, collecting samples from new and younger cohorts may be more scientifically productive than getting samples from aging cohorts. Work on DNA processing, metabolomics, and bioinformatics should continue to develop standardized methods while events are accruing in new cohorts. However, this ongoing methods work need not hinder concurrent biosample collection for later analysis. It will be important to collaborate more with biostatisticians when designing studies. Scientists from microbiology, epidemiology, bioinformatics, and statistics need to work together to develop reporting and data sharing standards that will ensure replication and reproducibility of studies. They should also work with journal editors to promulgate and employ these standards. This exciting new field holds great promise for improving our understanding of human health; more and regular engagement in these fields will accelerate our progress.

## Abbreviations

AHS-2: Adventist Health Study-2
API: Application Programmer Interface
BMI: Body mass index
COMPASS: Chicago Multiethnic Prevention and Surveillance Study
DACC: Data Analysis and Coordination Center
DCCPS: Division of Cancer Control and Population Sciences
DCEG: Division of Cancer Epidemiology and Genetics
EGRP: Epidemiology and Genomics Research Program
EQUATOR: (Enhancing the QUAlity and Transparency Of health Research) Network
FIT: Fecal immunochemical testing
FOBT: Fecal occult blood test
FTA: Flinders Technology Associates
GC/MS: Gas chromatography-mass spectrometry
HEALS: Health Effects of Arsenic Longitudinal Study
HPFS: Health Professionals Follow-up Study
ICCs: Intraclass correlation coefficient
IRP: Intramural Research Program
MAQC: Microarray quality control
MBQC: Microbiome quality control
MEB: Metabolic Epidemiology Branch
MEC: Multiethnic Cohort Study
MICRO-N: MICRObiome Among Nurses Study
NCI: National Cancer Institute
NHS-II: Nurses’ Health Study II
NIH: National Institutes of Health
NMI: National Microbiome Initiative
OTU: Operational taxonomic units
PCR: Polymerase chain reaction
QIIME 2: Quantitative Insights Into Microbial Ecology 2
RM: Reference materials
rRNA: ribosonal RNA
SCCS: Southern Community Cohort Study
S.E.: Standard error
SEQC: Sequencing Quality Control
SMHS: Shanghai Men’s Health Study
SNP: Single Nucleotide Polymorphisms
STROBE: Strengthening the Reporting of Observational Studies in Epidemiology
SWHS: Shanghai Women’s Health Study
VUMC: Vanderbilt University Medical Center’s
